# La Crosse virus, but not Jamestown Canyon virus, is dependent on the host translation termination factor eRF1 due to changes in the nonstructural protein NSm

**DOI:** 10.1128/mbio.01046-26

**Published:** 2026-06-15

**Authors:** Kaeri M. Medina, Sarah Dysinger, Rachel M. Braun, Swechha M. Pokharel, Trevor Griesman, David C. Schultz, Paul Bates, Sara Cherry

**Affiliations:** 1 Department of Pathology and Laboratory Medicine, University of Pennsylvania 6572, Philadelphia, Pennsylvania, USA; 2 Department of Microbiology, University of Pennsylvania 6572, Philadelphia, Pennsylvania, USA; 3 Department of Biochemistry and Biophysics, High Throughput Screening Core University of Pennsylvania 6572, Philadelphia, Pennsylvania, USA; Duke University School of Medicine, Durham, North Carolina, USA

**Keywords:** bunyavirus, california serogroup, translation, antiviral

## Abstract

Bunyaviruses are a class of segmented, negative-sense RNA viruses that are transmitted by arthropod and rodent vectors. Members of the *Orthobunyavirus* genus and California Serogroup (CSG) circulate in the U.S., including La Crosse Virus (LACV), which is a leading cause of pediatric encephalitis. The host factors required for infection, particularly those that impact bunyavirus translation, are poorly understood. We found that host eukaryotic release factor 1 (eRF1) is important for viral infection; genetic depletion or pharmacological degradation of eRF1 inhibits LACV infection. eRF1 is the only cellular translation termination factor that binds stop codons on mRNAs and promotes the release of the nascent peptide from the ribosome. Surprisingly, we found that not all bunyaviruses rely on eRF1: two distantly related bunyaviruses in the *Phenuiviridae* family, Rift Valley fever virus and Punta Toro virus, were not sensitive to eRF1 inhibition. In contrast, three closely related CSG viruses—Snowshoe hare virus, California Encephalitis virus, and Tahyna virus—are dependent on eRF1. This led us to test a more distantly related CSG virus, Jamestown Canyon virus, which is in a distinct subgroup, and found that it is not sensitive to eRF1 depletion. Genetic analysis revealed that the non-structural M protein (NSm), encoded on the M segment of LACV, conferred sensitivity to eRF1. Together, these data present a previously unknown molecular requirement for a subset of orthobunyaviruses and show that LACV NSm drives viral dependency on eRF1.

**IMPORTANCE:**

The California Serogroup (CSG) orthobunyavirus La Crosse virus (LACV) is the leading cause of arboviral pediatric encephalitis in the United States. Since there are no vaccines or therapeutics to treat LACV, we set out to identify host factors that promote infection as potential therapeutic targets. We found that host translation termination factor eRF1 is required for LACV infection and expected that this would be required for all bunyaviruses. While closely related CSG viruses were also dependent on eRF1, we found that eRF1 is dispensable for distantly related bunyaviruses. Surprisingly, Jamestown Canyon virus, another CSG virus, also does not depend on eRF1. This allowed us to use genetics to discover that the NSm protein sensitizes LACV to eRF1. This may represent a unique target for the treatment of LACV.

## INTRODUCTION

Bunyaviruses are a class of emerging and re-emerging viruses with a segmented negative, single-stranded RNA genome. They are globally distributed and spread by arthropod or rodent vectors (1). Many bunyaviruses can cause severe disease in humans, including encephalitis and hemorrhagic fever (2–4). Within the *Peribunyaviridae* family and *Orthobunyavirus* genus exists the California Serogroup (CSG), a subgroup of genetically and serologically related bunyaviruses spread by mosquito vectors, many of which circulate in the U.S. (3). Many CSG viruses are neurotropic and cause severe disease (5, 6). One species, La Crosse virus (LACV), is the leading cause of arboviral pediatric encephalitis in the United States (7, 8). Other CSG species found in the United States, such as Snowshoe hare virus (SSHV) and California Encephalitis virus (CEV), cause much lower incidence of disease. Although the phylogenetic and geographic differences among the CSG viruses are well documented, there are few studies that provide a mechanism for these phenotypic differences (3, 5, 6, 9). The geographic range of the vectors that spread the CSG viruses is increasing, and there is a lack of treatments. Understanding the molecular virology of these viruses is important for the development of new vaccines and therapeutics.

After viral entry into host cells, bunyaviruses release their segmented RNA genome into the cytoplasm, where the packaged RNA-dependent RNA polymerase (RdRp) begins transcribing the genomic segments into mRNAs for translation. Orthobunyaviruses are tri-segmented encoding small (S), medium (M), and large (L) segments (10). The S segment generates the nucleocapsid protein, required for viral RNA binding and replication, and nonstructural S protein (NSs), the main host innate immune antagonist (11). The M segment encodes a single polyprotein that is co-translationally cleaved by host proteases into the heterodimeric glycoproteins Gn/Gc, required for viral entry, and the nonstructural M protein (NSm), with unknown roles. The L segment generates the viral RdRp required for RNA transcription and replication. Little is known about the role of NSm during orthobunyavirus infection, especially for viruses within the CSG (2, 10, 12, 13).

Host mRNAs are transcribed in the nucleus, where they are capped on their 5′ end and polyadenylated at their 3′ end. Upon their export into the cytoplasm, the 5′ cap binds eukaryotic initiation factors that recruit the ribosome and initiate translation, while the polyA tail binds the polyA-binding protein (PABP) (14). Additionally, host translation termination is a tightly regulated process. When the translating ribosome encounters one of the three stop codons (UGA, UAG, and UAA), eRF1 is recruited to the ribosome, where it binds eukaryotic release factor 3 (eRF3) to form the eRF1/eRF3 termination complex (15–18). Upon binding the stop codon, eRF1 binds, eRF3 undergoes a conformational change that triggers peptide-tRNA hydrolysis to release the nascent peptide from the ribosome, and eRF3 interacts with PABP to promote ribosomal removal (19, 20). Bunyavirus mRNAs are synthesized in the cytoplasm, are capped but lack a polyA tail, and thus presumably cannot bind PABP, a critical host protein for mRNA stability, translation, and termination (1, 10, 21). This raises many questions regarding how bunyaviral mRNAs are stable and efficiently translated.

To identify host factors that impact bunyavirus infection, we performed a loss-of-function genetic screen, which revealed that eRF1 is required for LACV infection. Furthermore, we found that eRF3 is also required. eRF1 is necessary for translation termination; therefore, we expected all bunyaviruses to be sensitive to eRF1 depletion. We found that other closely related CSG viruses from the CEV complex (SSHV, CEV, and Tahyna virus [TAHV]) are dependent on eRF1. However, more distantly related bunyaviruses in the *Phenuiviridae* family (Rift Valley fever virus [RVFV] and Punta Toro virus [PTV]) are not. CSG viruses fall into three clades; we next tested Jamestown Canyon virus (JCV), a CSG virus that is in a distinct genetic clade (the Melao virus [MELV] complex) and found that JCV does not depend on eRF1. Genetic analysis revealed that the LACV M segment, and more specifically NSm, confers sensitivity to eRF1, uncovering a previously unknown role for bunyaviral NSm during infection (10, 13).

## RESULTS

### eRF1 is required for La Crosse virus infection

**Fig 1:**
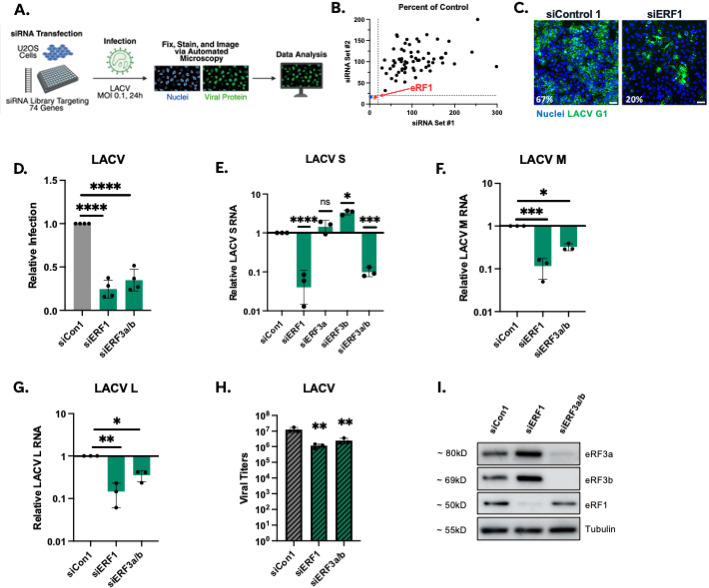
eRF1 is required for La Crosse virus infection. (**A**) Schematic of siRNA screen workflow. Human U2OS cells were transfected with siRNAs targeting 74 genes of interest from two independent suppliers and infected with LACV (LACV, MOI 0.1, 24 h). Cells were fixed and immunostained (infection, anti-LACV G1 and nuclei, Hoechst 33342). Automated imaging and image analysis were used to quantify cell number, number of infected cells, and percent infection. (**B**) Results of the screen were plotted as a percentage of the control for the percentage of infection. eRF1 depletion (red) decreases infection by >80% (dotted lines). (**C**) Representative images from the screen showing LACV infection in siControl 1 or siERF1 (LACV: green and nuclei: blue). Lower left: percent infected cells. Magnification 10×; scale bar is 200 µm. (**D–G**) U2OS cells were transfected with the indicated siRNAs for 72 h and infected with LACV (MOI 0.1) for 24 h. (**D**) Relative percent infection was measured by automated microscopy. Mean ± SD shown for *n* = 4. (**E**) LACV S RNA measured by RT-qPCR. Mean ± SD shown for *n* = 3. Comparing mean of each column with mean of control column (siCon1). (**F**) LACV M RNA measured by RT-qPCR. Mean ± SD shown for *n* = 3. (**G**) LACV L RNA measured by RT-qPCR. Mean ± SD shown for *n* = 3. GAPDH was used as a loading control gene for qPCR experiments. (**H**) U2OS cells were transfected with the indicated siRNAs for 72 h, infected with LACV (MOI 0.1) at 24 hpi, and supernatant was titered. Mean ± SD shown for *n* = 3. (**I**) U2OS cells were transfected with the indicated siRNAs for 72 h, and the indicated proteins were assessed by immunoblot. A representative immunoblot image is shown for *n* = 2. Dots represent individual experiments. Statistical analyses were performed using one-way analysis of variance with Dunnett’s multiple comparison test (**D–H**). *P < 0.05, **P < 0.01, ***P < 0.001, and ****P < 0.0001.

To identify host factors that impact LACV infection, we created a library of small interfering RNAs (siRNAs) to deplete 74 genes with a focus on RNA biology. We optimized an automated microscopy-based screen using human osteosarcoma U2OS cells and included siRNAs targeting anti-apoptotic RNAs (siDeath) as a positive control for knockdown efficiency (22, 23). We observed >90% cell death in siDeath-transfected cells compared to the negative control siRNA (siCon1; Fig. S1A). We screened this library for genes that play a role in LACV infection (1960 strain, multiplicity of infection (MOI) 0.1 for 24 h; Table S1). Using automated microscopy and automated image analysis, we quantified cell number (Hoechst, nuclei+) and percent infection (nuclei+, and LACV+) using an anti-LACV glycoprotein antibody (Fig. 1A). We identified three genes, *ETF1*, *SMU1*, and *EIF43A*, that, when depleted, led to a decrease in infection (≤20% infection; Fig. 1B and C). However, when we removed cytotoxic candidates (<50% cell viability), only *ETF1*, which encodes eRF1, remained as a candidate (Fig. S1B). We validated that eRF1 was depleted by the siRNAs at the RNA (Fig. S1C) and protein level (Fig. 1I; Fig. S1D). Next, we transfected cells with either siCon1 or siRNAs against eRF1 (siERF1), mock infected, or infected with LACV at MOI 0.1 for 24 h, and measured cell number and percent infection. Upon depletion of eRF1, there was a modest decrease in cell number (35% decrease, Fig. S1E) and a significant decrease in infection by automated microscopy (Fig. 1D). Next, we measured cellular levels of LACV S, M, and L viral RNAs by RT-qPCR and found that depletion of eRF1 led to a significant decrease in the levels of all three segments (Fig. 1E through G) (10, 24). Consistent with the impact on viral RNA, we observed a significant decrease in viral titers (Fig. 1H). We also tested whether LACV infection impacts eRF1 abundance and found that LACV infection did not impact the levels of eRF1 RNA or protein in the cell (Fig. S1F and G).

eRF1 binds eRF3 to form the eRF1/eRF3 termination complex, and thus, we tested whether eRF3 depletion impacts LACV infection. eRF3 has two isozymes, eRF3a and eRF3b, which are redundant (18). Individual depletion of eRF3a or eRF3b did not impact LACV RNA levels (Fig. 1E). However, the co-depletion of eRF3a and eRF3b led to a significant decrease in LACV percent infection and viral RNA of all three segments in the absence of cytotoxicity (Fig. 1D through G; Fig. S1E). We also found a significant reduction in viral titer with co-depletion of eRF3a and eRF3b (Fig. 1H). We validated co-depletion at the RNA level (Fig. S1H and I) and protein level (Fig. 1I). These data demonstrate that eRF1 and eRF3 are required for LACV infection.

Next, we conducted a time course to determine when eRF1 impacts LACV infection. We synchronized infection and measured viral RNA accumulation over time and observed no differences in early time points (4 h and 6 h) (25). Only at 8 h post-infection did we observe a significant decrease in viral RNA, suggesting that eRF1 was acting at an early post-entry step (Fig. S2A).

Bunyavirus genomic transcription is coupled to translation; viral mRNA is transcribed by the RdRp, while the nascent mRNA is simultaneously translated by host ribosomes (26). It is possible that depletion of eRF1 could prevent translation termination and lead to the accumulation of double-stranded RNA (dsRNA), as the genomic transcript and the nascent mRNA are complementary (27). To test this, we depleted eRF1 in LACV-infected cells and stained for dsRNA (28). As a positive control, we infected with the flavivirus West Nile virus (WNV), which produces dsRNA during its replication cycle (29, 30). We observed accumulation of dsRNA in the WNV-infected cells, while control or eRF1-depleted LACV-infected cells showed no accumulation of dsRNA (Fig. S2B). This suggests that loss of eRF1 does not induce dsRNA production during LACV infection. It is also possible that depletion of eRF1 impacts the stability of viral RNAs since loss of eRF1 is known to destabilize mRNAs in mammalian cells (31). To test this, we infected siERF1-depleted cells with LACV before treating with cycloheximide to block new translation. Blocking viral translation is known to prevent new genome transcription (32). We synchronized the infection of either control or eRF1-depleted cells and treated with cycloheximide at 18 hpi. We then measured viral RNA over time and observed decay of viral RNA in control cells and found no difference in the rate of LACV RNA decay upon depletion of eRF1 (Fig. S2C). Altogether, these data suggest that eRF1 is required post-entry but does not impact viral RNA stability.

eRF1/eRF3 is also a part of the SMG-1-UPF1-eRF1-eRF3 (SURF) complex, which promotes the degradation of host mRNAs by triggering nonsense-mediated decay (NMD) (33, 34). eRF1 may impact LACV infection through its role in the SURF complex. To test this, we employed two complementary approaches. First, we treated U2OS cells with the NMD inhibitor NMDI14, found that there was no cytotoxicity (Fig. S3A), and confirmed on-target activity by monitoring the cellular NMD target SC35, which increased upon NMDI14 treatment (Fig. S3B) (35, 36). Upon NMD inhibition, we found no decrease of LACV infection as measured by automated microscopy (Fig. S3C). We also utilized siRNAs to deplete NMD factors UPF1, UPF2, and UPF3A (33, 37). We confirmed target depletion and NMD inhibition (Fig. S3D through G). Again, we found that inhibition of NMD did not impact LACV infection as measured by automated microscopy (Fig. S3H). Together, these data suggest that eRF1 is not impacting LACV infection through its role in the SURF complex and is instead acting through its role as a translation termination factor or another novel, unknown function.

### Treatment with an eRF1 degrader blocks LACV infection

**Fig 2:**
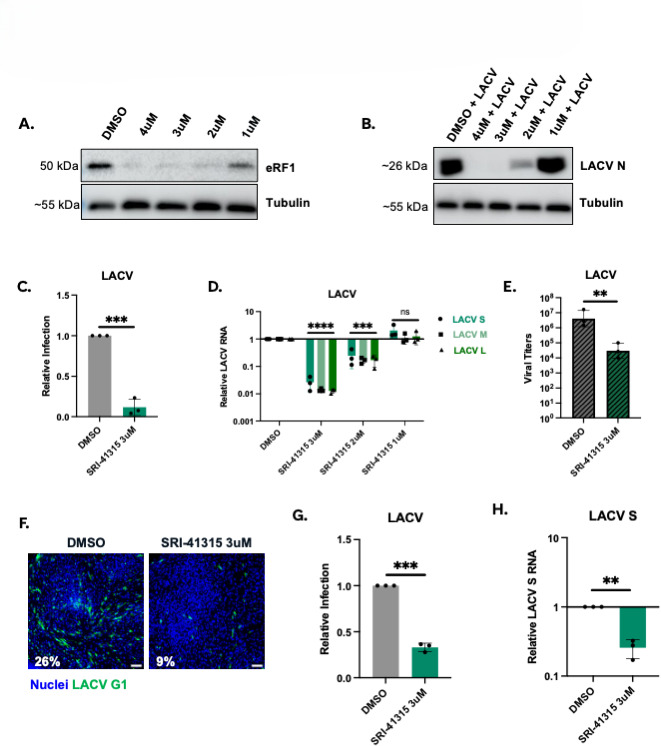
Treatment with an eRF1 degrader inhibits LACV infection. (**A**) U2OS cells were treated with vehicle or the indicated concentrations of SRI-41315 for 20 h. eRF1 levels were measured by immunoblot with tubulin as a loading control. A representative immunoblot is shown for *n* = 2. (**B–D**) U2OS cells were treated with vehicle or the indicated concentrations of SRI-41315 for 20 h and infected with LACV (MOI 0.1) for 24 h. (**B**) LACV nucleocapsid protein levels were assessed by immunoblot with tubulin as a loading control. A representative immunoblot image is shown for *n* = 2. (**C**) Relative percent infection was measured by automated microscopy. Mean ± SD shown for *n* = 3. (**D**) LACV S, M, and L RNA was measured by RT-qPCR. Mean ± SD shown for *n* = 3. GAPDH was used as a loading control. (**E**) U2OS cells were treated with vehicle or 3 µM of SRI-41315 for 20 h, infected with LACV (MOI 0.1) for 24 h, and supernatant was titered. Mean ± SD shown for *n* = 3. (**F–H**) Human foreskin fibroblasts (HFFs) were treated with DMSO or SRI-41315 at 3 µM for 20 h and infected with LACV (MOI 1) for 24 h. (**F**) Representative images shown (LACV: green and nuclei: blue). Magnification 10×; scale bar is 200 µm. Lower left: percent infected cells. (**G**) Relative percent infection was measured by automated microscopy. Mean ± SD shown for *n* = 3. (**H**) LACV S RNA was measured by RT-qPCR with 18S as a loading control. Mean ± SD shown for *n* = 3. Dots represent individual experiments. Statistical analyses were performed using Student’s (unpaired, two-tailed) *t*-test (**C, G, and H**), and two-way analysis of variance with Dunnett’s multiple comparison test (**D**), and a lognormal Welch’s *t*-test (**E**). **P < 0.01, ***P < 0.001, ****P < 0.0001, and ns = not significant.

SRI-41315 is a small molecule that induces the ubiquitylation and subsequent proteasomal degradation of eRF1 (38). We tested whether degradation of eRF1 by SRI-41315 would phenocopy siRNA-mediated depletion of eRF1. We found that treatment with 4 µM, 3 µM, and 2 µM SRI-41315 for 20 h leads to loss of eRF1 protein (Fig. 2A) (39). Next, we treated cells with vehicle (DMSO) or SRI-41315, infected with LACV, and found that SRI-41315 treatment resulted in a significant decrease of LACV nucleocapsid protein (Fig. 2B). We also found that treatment with SRI-41315 at 3 µM had a significant impact on LACV infection by automated microscopy (Fig. 2C). Treatment with SRI-41315 at 3 µM did have a modest impact on cell viability (~20%), but not at 2.5 µM or lower (Fig. S1J). We measured the impact of SRI-41315 on LACV viral RNA by RT-qPCR and observed striking decreases at both 3 µM and 2 µM (Fig. 2D). SRI-41315 treatment also significantly reduced LACV titers (Fig. 2E).

We tested the role of eRF1 in primary human foreskin fibroblasts (HFFs) and found that treatment with SRI-41315 at 3 μM led to a significant decrease in LACV infection by automated microscopy with no impact on cell viability (Fig. 2F and G; Fig. S1K). SRI-41315 treatment also reduced LACV RNA by ~10-fold (Fig. 2H). Altogether, these data demonstrate a role for host translation termination machinery during LACV infection.

### eRF1 is required for infection of California Serogroup bunyaviruses but not Phenuiviridae viruses

**Fig 3:**
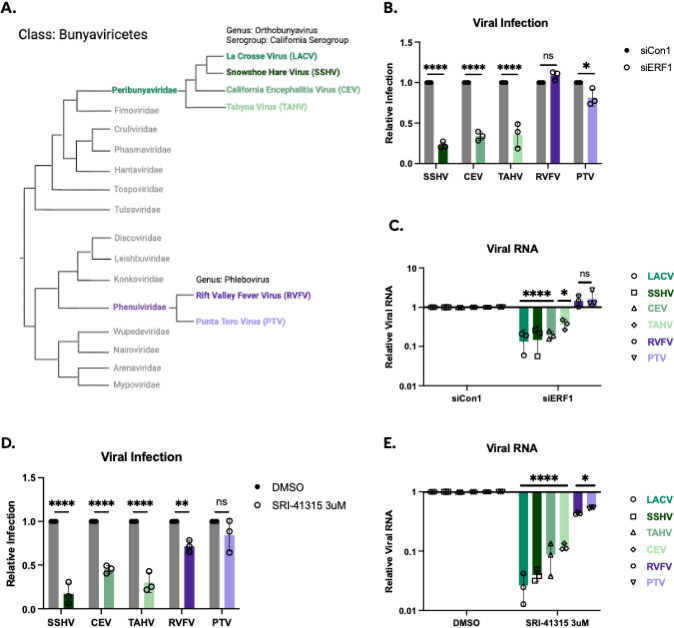
eRF1 is required for infection of California serogroup bunyaviruses but not Phenuiviridae viruses. (**A**) *Bunyaviricetes* phylogenetic tree based on large (**L**) protein amino acid sequences of the 15 families. Tree segments are not to scale. *Peribunyaviridae* (green) and *Phenuiviridae* (purple) other viral families (gray). (**B and C**) U2OS cells were transfected with siCon1 or siERF1 for 72 h. Cells were infected with SSHV (MOI 5), CEV (MOI 15), TAHV (MOI 1), RVFV (MOI 1), or PTV (MOI 15) for 24 h. (**B**) Relative percent infection was measured by automated microscopy. Mean ± SD shown for *n* = 3. (**C**) Viral RNA was measured by RT-qPCR with GAPDH as a control. Mean ± SD shown for *n* = 3. (**D and E**) U2OS cells were treated with vehicle or 3 μM of SRI-41315 for 20 h. Cells were infected with SSHV (MOI 0.1), CEV (MOI 15), TAHV (MOI 0.05), RVFV (MOI 1), or PTV (MOI 15) for 24 h. (**D**) Relative percent infection was measured by automated microscopy. Mean ± SD shown for *n* = 3. (**E**) Viral RNA was measured by RT-qPCR with GAPDH as a control. Mean ± SD shown for *n* = 3. Dots represent individual experiments. Statistical analyses were performed using a two-way analysis of variance with Šídák’s correction for multiple comparisons (**B–E**). *P < 0.05, ****P < 0.0001, and ns = not significant.

Given the dependence of LACV on eRF1, we evaluated the importance of eRF1 in the replication of additional bunyaviruses. We tested RVFV and PTV, which both belong to the distantly related *Phenuiviridae* family and *Phlebovirus* genus (Fig. 3A) (12). We found that depletion of eRF1 had no impact on RVFV infection and only a minor impact on PTV infection as measured by automated microscopy (Fig. 3B). We also measured the levels of viral RNA and found that eRF1 depletion had no impact on RVFV and PTV replication (Fig. 3C). This suggests that not all bunyaviruses are dependent on eRF1 for replication. LACV belongs to the CSG, within the *Peribunyaviridae* family and *Orthobunyavirus* genus (Fig. 3A) (6). We tested three additional CSG viruses: SSHV, CEV, and TAHV (Fig. 3A). We found that depletion of eRF1 led to significant decreases in infection of all three viruses as measured by automated microscopy and RT-qPCR (Fig. 3B and C).

We further tested the requirement for eRF1 using SRI-41315. As observed with siRNA-mediated depletion, we found that treatment with SRI-41315 had little impact on RVFV and no impact on PTV, but significantly impacted SSHV, CEV, and TAHV as measured by automated microscopy (Fig. 3D). Furthermore, SRI-41315 treatment significantly reduced SSHV, CEV, and TAHV RNA, but had only a modest impact on RVFV or PTV RNA (Fig. 3E). These data further support a specific role for eRF1 in CSG infection, but not RVFV and PTV, showing that eRF1 is only required for infection of some bunyaviruses.

The sensitivity of four closely related CSG viruses to eRF1 depletion indicates that these viruses have a distinct dependency that drives this phenotype. One major difference between the *Orthobunyaviruses* and *Phleboviruses* is their differential S segment coding strategies (12). *Phleboviruses* have an ambisense coding strategy on the S segment that produces two mRNAs, one from their genome and the other from the antigenome (Fig. S4A). In contrast, the CSG viruses’ S segment produces one mRNA that encodes both N and NSs via two overlapping open reading frames (ORFs; Fig. S4A). This coding difference could be responsible for the differential eRF1 dependencies between these genera of viruses. To test this, we obtained an LACV virus with a mutated start codon in the NSs ORF (LACV ∆NSs) so that only N can be produced (Fig. S4A) (11, 40). NSs is the major immune antagonist; we first verified that LACV ∆NSs displayed an impaired interferon (IFN) response by measuring the expression of the IFN-stimulated gene IFIT1 by automated microscopy (41). As expected, we observed a greater percentage of IFIT1-positive cells during LACV ∆NSs infection when compared to LACV infection (Fig. S4B). Next, we infected control or eRF1-depleted cells and measured infection by automated microscopy and viral RNA by RT-qPCR. We found that eRF1 depletion resulted in a significant decrease in LACV ∆NSs infection in both assays, indicating that the differential S segment genomic coding strategy is not responsible for viral dependency on eRF1 (Fig. S4C and D).

### Jamestown Canyon virus, a CSG virus in the MELV complex, does not depend on eRF1

**Fig 4:**
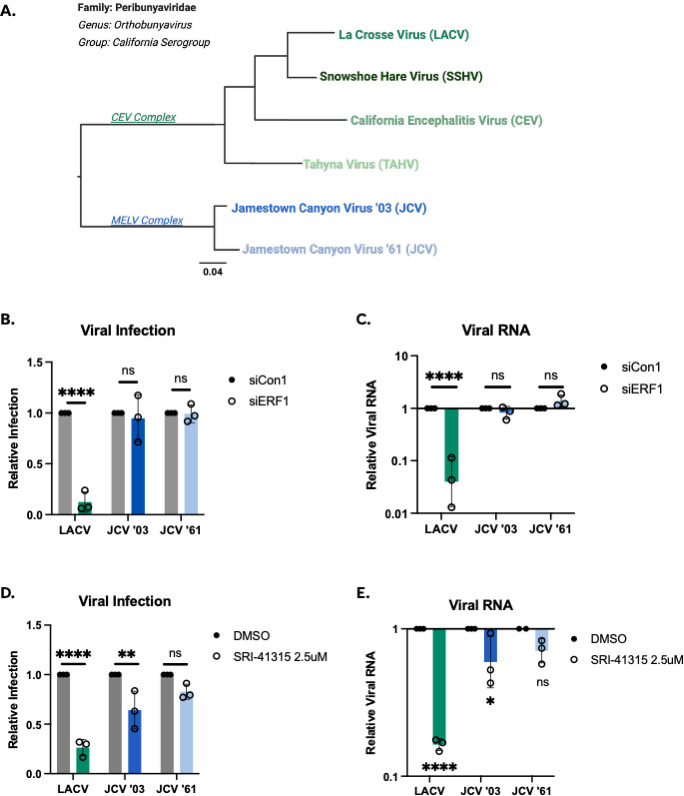
Jamestown Canyon virus, a CSG virus in the MELV complex, does not depend on eRF1. (**A**) Phylogenetic tree of LACV, SSHV, CEV, TAHV, and JCV assembled based on the M segment amino acid sequence. Tree branches to scale: 0.04 indicates the mean number of substitutions per site for the length of the bar. CEV complex viruses are in green. MELV complex viruses are in blue. (**B and C**) U2OS cells were transfected with siCon1 or siERF1 for 72 h. Cells were infected with LACV (MOI 0.1), JCV ’03 (MOI 5), or JCV ’61 (MOI 5) for 24 h. (**B**) Relative percent infection was measured by automated microscopy. Mean ± SD shown for *n* = 3. (**C**) Viral RNA was measured by RT-qPCR with GAPDH as a control. Mean ± SD shown for *n* = 3. (**D and E**) U2OS cells were treated with DMSO or SRI-41315 2.5 µM for 20 h. Cells were infected with LACV (MOI 0.1), JCV ’03 (MOI 5), or JCV ’61 (MOI 5) for 24 h. (**D**) Relative percent infection was measured by automated microscopy. Mean ± SD shown for *n* = 3. (**E**) Viral RNA measured by RT-qPCR with GAPDH as a control. Mean ± SD shown for *n* = 3. Dots represent individual experiments. Statistical analyses were performed using a two-way analysis of variance with Šídák’s correction for multiple comparisons (**B–E**). *P < 0.05, **P < 0.01, ****P < 0.0001, and ns = not significant.

The CSG viruses fall into three distinct clades: the CEV complex, MELV complex, and the Trivittatus virus (TVTV) complex (3, 6). LACV, SSHV, CEV, and TAHV all belong to the CEV complex, while another CSG virus, Jamestown Canyon virus (JCV), is in the MELV complex (Fig. 4A). Like LACV, JCV also circulates in the United States and can cause encephalitis but does so across a wide age range (42, 43). We tested the sensitivity of two JCV isolates, JCV ’61 and JCV ’03, to eRF1 depletion and observed no significant inhibition of infection as measured by automated microscopy or RT-qPCR upon siRNA depletion of eRF1 (Fig. 4B and C) (44). Treatment with SRI-41315 had a modest impact on JCV ’03 infection and no impact on JCV ’61 infection by automated microscopy and RT-qPCR (Fig. 4D and E). We also tested the impact of NMD inhibition on JCV ’61 and saw no decrease in infection by automated microscopy (Fig. S5A and B). These data suggest that there is a genetic difference that accounts for their differential dependency on eRF1.

### The LACV M segment is required for viral sensitivity to eRF1

**Fig 5:**
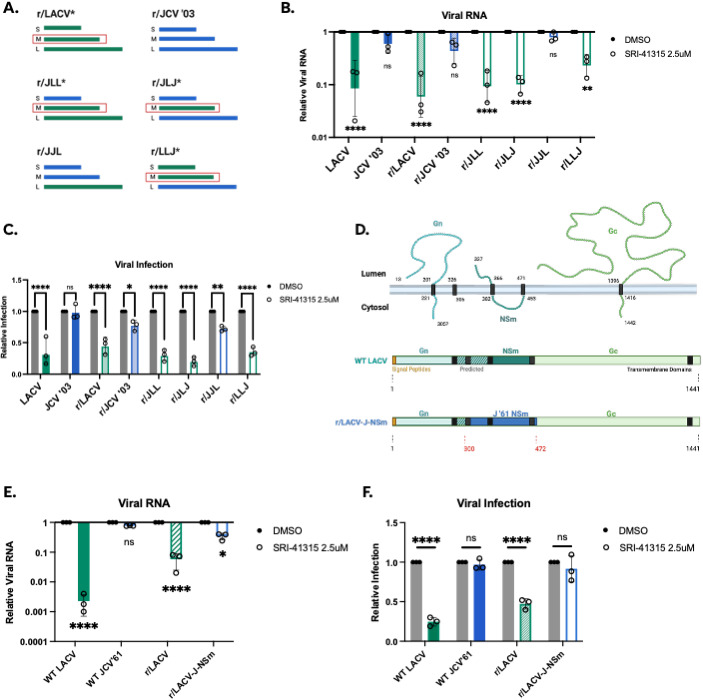
LACV NSm confers sensitivity to eRF1. (**A**) Schematic of the recombinant viruses. LACV segments in green, and JCV ’03 segments in blue. Asterisk denotes the viruses sensitive to SRI-41315 treatment. LACV M segments boxed in red. (**B and C**) U2OS cells were treated with DMSO or SRI-41315 2.5 µM for 20 h. Cells were infected with WT LACV (MOI 0.5), WT JCV ‘03 (MOI 1), r/LACV (MOI 0.05), r/JCV ‘03 (MOI 0.5), r/JLL (MOI 0.01), r/JLJ (MOI 0.005), r/JJL (MOI 0.5), and r/LLJ (MOI 0.85). (**B**) Viral RNA measured by RT-qPCR with GAPDH as control. Mean ± SD shown for *n* = 3. (**C**) Relative percent infection was measured by automated microscopy. Mean ± SD shown for *n* = 3. (**D**) Schematic of LACV M polyprotein topology at the ER membrane, based on DeepTMHMM analysis. Schematic of the LACV M segment and the LACV-J-NSm M segment: LACV M segment has its NSm region replaced with the predicted NSm region from JCV ’61 in blue. Amino acids replaced shown in red. The signal peptide is shown in yellow, and transmembrane domains are in black. The white hatched region indicates unknown coding regions between Gn and NSm. (**E and F**) U2OS cells were treated with DMSO or SRI-41315 2.5 µM for 20 h. Cells were infected with LACV (MOI 0.5), JCV ‘61 (MOI 5), r/LACV (MOI 1), and r/LACV-J-NSm (0.375) for 24 h. (**E**) Viral RNA measured by RT-qPCR with GAPDH as a control. Mean ± SD shown for *n* = 3. (**F**) Relative percent infection was measured by automated microscopy. Mean ± SD shown for *n* = 3. Dots represent individual experiments. Statistical analyses were performed using a two-way analysis of variance with Šídák’s correction for multiple comparisons (**B, C, E, and F**). *P < 0.05, **P < 0.01, ***P < 0.001, ****P < 0.0001, and ns = not significant.

Orthobunyaviruses readily reassort, and we used this strategy to define which segment of LACV is required for eRF1 dependence (45, 46). We tested six recombinant viruses: LACV virus which encodes all three LACV segments (r/LACV); the JCV ‘03 virus (r/JCV‘03) which encodes all three JCV ‘03 segments; as well as the combinations with JCV S, LACV M, and LACV L (r/JLL); JCV S, LACV M, and JCV L (r/JLJ); JCV S, JCV M, and LACV L (r/JJL); and LACV S, LACV M, and JCV L (r/LLJ; Fig. 5A). These viruses were confirmed to infect U2OS cells similarly as measured by viral titer (Table S2). We then measured the impact of eRF1 inhibition on this panel of viruses. As expected, treatment with SRI-41315 resulted in a significant decrease in WT LACV and r/LACV infection and had no impact on WT JCV ‘03 or r/JCV ‘03 infection as measured by RT-qPCR (Fig. 5B). Furthermore, we found that SRI-41315 treatment significantly impacted r/JLL and r/LLJ, indicating that the S and L segments of LACV are dispensable for sensitivity to eRF1 inhibition. In contrast, r/JLJ, a virus with the S and L segments from JCV and the M segment from LACV, was sensitive to SRI-41315 treatment (Fig. 5B). Conversely, treatment with SRI-41315 had no impact on r/JJL, a virus with the S and M segments from JCV and the L segment from LACV (Fig. 5B). Treatment with SRI-41315 also significantly reduced WT LACV, r/LACV, r/JLL, r/JLJ, and r/LLJ infection as measured by automated microscopy. SRI-41315 treatment only modestly impacted r/JCV ’03 and r/JJL (~25% reduction; Fig. 5C). Altogether, these data demonstrate that the M segment of LACV encodes the sensitivity to eRF1.

### LACV NSm confers sensitivity to eRF1

The LACV M segment encodes a single polyprotein that is embedded in the ER membrane, where it is processed into the structural glycoproteins Gn and Gc and NSm (Fig. 5D) (10). Gn and Gc are type I integral transmembrane proteins that heterodimerize in the ER, while NSm is predicted to be a transmembrane protein (13, 47). Gn/Gc promotes entry, while little is known about the function of NSm. Since the eRF1 dependency involves intracellular replication, not entry, we hypothesized that the LACV NSm confers sensitivity to eRF1. The exact boundaries between the Gn-NSm-Gc proteins are unclear, and the host proteases and cleavage sites are only known for orthobunyaviruses that do not belong to the CSG (13, 48). Using previous studies and protein topology prediction software (DeepTMHMM), we analyzed the transmembrane domains, topology, and size of NSm and found a large domain that is predicted to be cytoplasmic (Fig. 5D; Table S3) (10, 13, 47, 49–51). As JCV’61 is least sensitive to SRI-41315, it was selected for further study (Fig. 4D). We generated a recombinant LACV that encodes NSm from JCV ’61 (r/LACV-J-NSm) and tested sensitivity to eRF1 depletion (Fig. 5D). As expected, treatment with SRI-41315 resulted in a significant decrease in WT LACV and r/LACV infection and had no impact on WT JCV ’61 infection as measured by microscopy or RT-qPCR (Fig. 5E and F). In contrast, LACV encoding JCV NSm was resistant to eRF1 depletion. While SRI-41315 treatment led to a modest decrease in viral RNA by RT-qPCR, we measured no impact on r/LACV-J-NSm infection by automated microscopy. This indicates that NSm sensitizes LACV to eRF1 (Fig. 5E and F). Altogether, we have identified a novel role for LACV NSm in altering viral dependence on eRF1.

## DISCUSSION

Bunyaviruses are an important class of segmented, negative-sense RNA viruses that can cause diverse pathogenesis in humans with no treatment options (52–54). The CSG viruses are widespread, and LACV is the leading cause of pediatric arboviral encephalitis in the United States (8, 55). We discovered that LACV and three other CEV complex viruses (SSHV, CEV, and TAHV) depend on eRF1 during infection (3). Since eRF1 is generally important in translation termination, we hypothesized that it would impact the infection of all bunyaviruses. However, we found that bunyaviruses from the *Phenuiviridae* family and *Phlebovirus* genus, RVFV and PTV, did not depend on eRF1. To narrow down the requirement for eRF1, we next tested JCV and found that eRF1 was dispensable (3). The CSG viruses have been traditionally distinguished by phylogenetic studies, differing pathologies, and transmission strategies between their hosts and vectors (3, 5, 6, 9, 56). Here, we identify a molecular difference in host factor dependency for viruses within the CSG (57).

We mapped viral sensitivity of eRF1 to NSm within the M segment (10, 13). While Gn/Gc have well-studied functions in viral entry, the role of NSm, in particular for CSG viruses, is poorly understood (12, 45, 58–61). RVFV NSm displays anti-apoptotic activity by suppressing caspase activity, regulates the p38 MAPK stress response, and can be found at the ER, mitochondrial membrane, and cytoplasm (12, 62–68). In mammalian cells, the distantly related orthobunyavirus Bunyamwera virus (BUNV) NSm co-localizes with Gn/Gc in the Golgi and contributes to the formation of tubular structures, potentially aiding in viral assembly (13, 69, 70). The N-terminal domain of BUNV NSm is required for maturation of Gn/Gc, while the C terminus was shown to function as an internal signal sequence for the Gc glycoprotein (58). For Schmallenberg virus, another distantly related orthobunyavirus, NSm also plays a role in localizing the Gc glycoprotein to the Golgi body (71). In mosquitoes, RVFV NSm and BUNV NSm are critical for infection and dissemination from the mosquito midgut, making it vital for propagation in its mosquito vector (59, 62, 63). For LACV, NSm plays a role in stabilizing and promoting high levels of infection in *Aedes triseriatus* mosquitoes, although the mechanism is unknown (60). Thus, the discovery that LACV NSm mediates viral sensitivity to eRF1 is unexpected.

eRF1 has a stop codon preference of UAA > UAG >> UGA, where UAA promotes efficient termination (14, 72). Additionally, the nucleotides upstream from the stop codon, at the +4 and +8 positions, also play a role (73). It is possible that LACV NSm requires eRF1 to enhance the termination efficiency of its own mRNAs and that JCV does not depend on this interaction. We analyzed the stop codon usage of LACV, SSHV, CEV, TAHV, JCV ’03, and JCV ’61, and found no obvious pattern that distinguishes the CEV complex viruses and the JCV isolates (Table S4). Therefore, the mechanism by which eRF1 and LACV NSm cooperate to facilitate LACV infection is unclear. It is still likely that LACV depends on eRF1 to ensure efficient termination. The ER is a privileged site of protein synthesis; ribosomes at the ER can translate mRNAs without signal peptides and remain functional even when PABP is inactivated (74). Additionally, NSm has been described as an integral membrane protein, and it may be subject to further processing for release into the cytosol (13). Local translation and processing of the M polyprotein at the rough ER may enable NSm to recruit eRF1 for preferential, and therefore efficient, termination of viral mRNAs.

Our data suggest that NSm in the CEV complex viruses confers sensitivity to eRF1. There are 18 orthobunyaviruses classified into three complexes, and thus, we aligned the available NSm sequences of 16 CSG viruses (Fig. S6). At the edge of the cytosolic region, we identified a conserved asparagine at residue 453 across the CEV complex that is an aspartic acid or leucine in four MELV viruses (JCV ’03, JCV ’61, INKV, and JSV) and all three TVTV viruses. This change could alter the dependence on eRF1 by impacting interactions with the host protein or NSm cleavage (Fig. S6 and S7) (75, 76). If so, the subset of MELV viruses (SAV, LUMV, KEYV, MELV, SDNV, and SORV) that have an asparagine at this position may also be sensitive to eRF1 depletion. Further studies are needed to determine if these viruses are dependent on eRF1, if this residue is responsible for this phenotype, and the exact mechanism by which sensitive CSG viruses utilize eRF1 during infection.

Altogether, we have defined a new role for bunyavirus NSm and a link between LACV NSm and translation termination factor eRF1 in mammalian cells. We have also demonstrated a key molecular difference among the CSG bunyaviruses in their sensitivity to translation termination machinery and defined a role for NSm in facilitating this dependence for LACV.

## MATERIALS AND METHODS

### Experimental model details

#### Cells, viruses, and reagents

U2OS (ATCC, Htb-96), VeroE6 (ATCC, CRL-1586), and BHK-21 (ATCC, CCL-10) cells were maintained as described (28). HFF cells (ATCC and SCRC-1041) were maintained in Dulbecco’s modified Eagle’s medium (DMEM, Gibco) supplemented with 10% FBS, penicillin/streptomycin (Gibco), MED199Earles (Invitrogen), and Gentamicin at 37°C/5% CO_2_. All cell lines were confirmed to be mycoplasma negative. LACV 1960, CEV BFS-283, and PTV Balliet were obtained from BEI resources. RVFV MP12 was a gift from Dr. Michael Diamond (WashU). LACV ∆NSs was provided by Dr. Kellie Ann Jurado (UPenn), with permission from Dr. Friedemann Weber (Jutus-Liebig University, Germany). SSHV Montana 1959, TAHV Czechoslovakia ‘92, JCV 2003, and JCV 1961 were provided by Dr. Paul Bates (UPenn). LACV, RVFV, SSHV, and TAHV were propagated in BHK, CEV in C6/36, PTV in LLC-MK2, and JCV ’03 and JCV ‘61 in Vero E6 cells. Viral titers were determined by tissue culture infectious dose (TCID_50_) using BHK or Vero E6 cells. The following primary antibodies were used: anti-LACV G1 (S. Soldan, Wistar), anti-LACV N (Novus NBP2-41225), anti-RVFV Gn/Gc (4D4), CEV immune ascitic fluid (ATCC VR-1213AF), PTV immune ascitic fluid (ATCC VR-1225AF), anti-WNV 4G2 (M. Diamond, WashU), eRF1 (Cell Signaling, #13916S), eRF3a (Proteintech, #10763-1-AP), eRF3b (Proteintech, #12989-1-AP), α-tubulin (T6199, Sigma), Anti-dsRNA J2 (Millipore, #MABE1134), and anti-IFIT1 antibody (Cell Signaling Technology #14769). We utilized the following secondary antibodies: AlexaFluor488, AlexaFluor594, Hoechst 33342, and HRP-conjugated secondary antibodies (α-mouse or α-rabbit; Amersham). SRI-41315 (MedChem Express HY-150059) and NMDI14 (Selleck Chemicals E1672) were diluted in DMSO and used at the indicated concentrations.

### Recombinant viruses

The pT7ribo-LACV-cSnoEco (pLACV-S, S segment), pT7ribo-LACV-cMPro (pLACV-M, M segment), and pT7ribo-LACV-cLPro (pLACV-L, L segment) plasmids were provided by Dr. Kellie Ann Jurado with permission from Friedmann Weber (40). The JCV’03 infectious clone system was generated by cloning cDNA of the complete JCV’03 S, M, and L segments into a T7-driven backbone (44, 77). To generate the r/LACV-J-NSm plasmid, the JCV ’61 NSm sequence was cloned from infected cell RNA amplified from cDNA by PCR. Insert and backbones were assembled using NEBuilder HiFi DNA Assembly Master Mix (NEB M5520AA). All primers used are provided in Table S5. Insertion of the desired sequence was confirmed by Sanger sequencing. All recombinant viruses (r/LACV, r/JCV ’03, r/JLL, r/JLJ, r/JJL, r/LLJ, and r/LACV-J-NSm) were generated by transfecting BHK BSR-T7/5 cells with 1 µg of each plasmid (pLACV-S, pLACV-M, pLACV-L, pJCV-S, pJCV-M, pJCV-L, and pLACV-J-NSm), 1 µL Lipofectamine 3000 (Thermo Fisher Scientific), and 1 µL P3000 reagent per µg of total DNA. Transfected cells were incubated for 3–5 days, and then supernatants were collected, and cells were detached by trypsinization. Cells were pooled with the supernatant, growth medium, and Vero E6 cells and plated in 10 cm dishes. Once substantial CPE was observed, and passage 0 viral stocks were harvested, clarified, and aliquoted as passage 0 (P0) virus stocks. Passage 1 (P1) stocks were generated by amplification on Vero E6 cells for experiments. All virus work, including recombinant viruses, was approved by the Institutional Biosafety Committee at the University of Pennsylvania.

### Method details

#### siRNA transfections

U2OS cells were transfected using HiPerfect (Qiagen) or RNAiMAX (Thermofisher) at 20 nM for 72 h. Pooled siRNAs were used unless noted. Gene-specific siRNAs are from Sigma; siControl 1 from Thermo Scientific (AM4635) and AllStars Hs Cell Death siRNA from Qiagen (#1027299) were used as controls. All siRNA gene targets are in Table S6.

#### High-throughput RNAi screening and image analysis

Pooled siRNAs targeting 74 genes (Table S1) were transfected into U2OS cells for screening as previously described (28). Infected cells were stained with anti-LACV G1 and 10 µg/mL Hoechst 33342 and imaged at 10× (ImageXpress Micro, Molecular Devices) with nine sites per well. The total number of cells (nuclei+) and the number of infected cells (LACV+) were measured using the cell scoring algorithm (MetaXpress 6.7.0), and the percentage of infected cells (LACV + cells/nuclei count) per well was calculated. siRNA wells were normalized to aggregated siCon wells and expressed as Percentage of Control (POC = [%Infection_sample siRNA_/ Average %Infection_control siRNA_] × 100) in Spotfire (PerkinElmer).

#### RNA isolation, reverse transcription, and quantitative reverse transcription PCR

Total RNA was extracted, and RT-qPCR was performed as previously described (28). Primer sequences used are in Table S5.

#### Immunoblots

Total protein was collected in RIPA buffer and analyzed by SDS/PAGE gel as previously described (28).

#### Viral titer quantification

Titer was quantified from siRNA or drug-treated U2OS cells infected with LACV (MOI 0.1) as previously described (28, 78, 79).

#### Phylogenetic analysis

The *Bunyaviricetes* Class and CSG trees are based on the large and medium protein, respectively (80–82). Trees were built with BV-BRC.org Protein Tree Builder using MAFFT alignment, FastTree, and LG parameters (83). Representative species for each family were selected based on a previous study (84). The resulting trees were adapted in Biorender. Original trees are in the supplementary figures (Fig. S8A and B). GenBank accession numbers are in Table S7.

#### DeepTMHMM M protein analysis

The LACV and JCV ’61 M protein topology was predicted using DeepTMHMM and GenBank sequences (Table S3) (51). These predictions were utilized for Fig. 5D and to inform the NSm boundaries for the r/LACV-J-NSm virus.

### Confocal microscopy

For dsRNA analysis, U2OS cells were seeded on coverslips, infected with LACV or WNV, fixed, and processed for fluorescent microscopy (Leica TCS SPE-II with 63× objective as previously described [28]).

#### RNA stability assay

Control or eRF1*-*depleted cells were infected for 18 h with LACV, MOI 0.1 followed by the addition of 50 µg/mL of cycloheximide. At each time point, RNA was collected for downstream extraction and qPCR analysis.

#### JalView protein alignments

The predicted NSm sequences from 16 CSG viruses were imported into JalView (Table S7) and amino acids colored using the Clustal X color scheme (85).

#### AlphaFold

We imported amino acids 371–471 from LACV M polyprotein into AlphaFold Version 3. The resulting structure was exported (Fig. S7) (86).

### Quantification and statistical analysis

Automated microscopy, RT-qPCR, and TCID_50_ data were compiled and analyzed using Prism Version 11 (GraphPad). All replicate experiments are independent biological experiments. All statistics performed on RT-qPCR data were log_2_ transformed prior to analysis. The specific tests and numbers of experiments are indicated in the Figure legends. Statistical significance was assigned when *P* values were <0.05.

## SUPPLEMENTAL MATERIAL

Supplemental Information mbio.01046-26-s0001.pdf Supplemental figures and tables.

ASM does not own the copyrights to Supplemental Material that may be linked to, or accessed through, an article. The authors have granted ASM a non-exclusive, world-wide license to publish the Supplemental Material files. Please contact the corresponding author directly for reuse.
