# Supplementary material for: La Crosse virus, but not Jamestown Canyon virus, is dependent on the host translation termination factor eRF1 due to changes in the nonstructural protein NSm
Source: mBio. 2026 Jun 15;17(7):e01046-26. doi: 10.1128/mbio.01046-26 (PMC13343935; doi:10.1128/mbio.01046-26)
Supplement: Supplemental Information — Supplemental figures and tables. [file mbio.01046-26-s0001.pdf]

Supplementary Figure 1: Cell Viability and Knockdown Validation

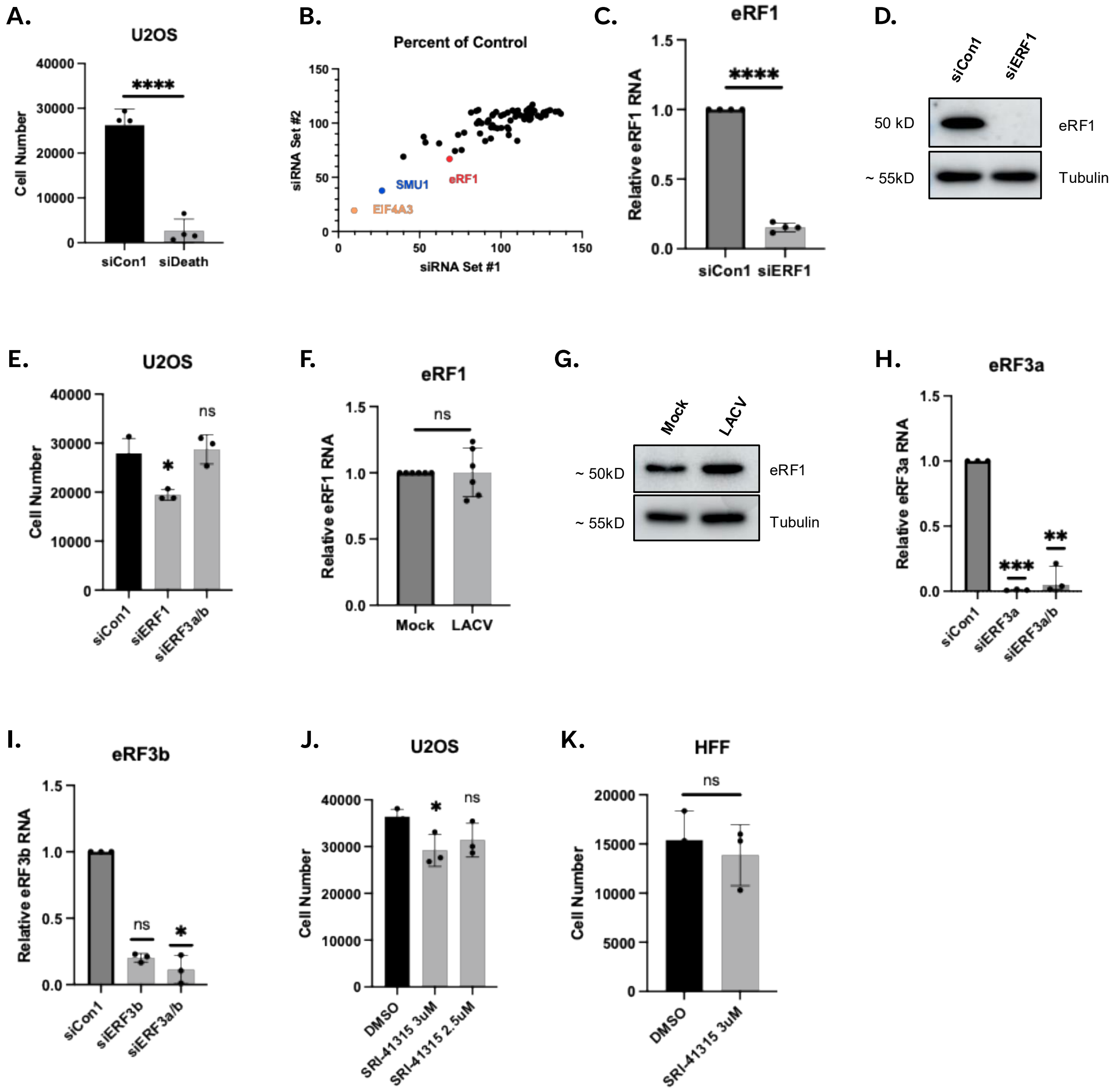

Supplementary Figure 2: eRF1 Depletion Does Not Induce LACV RNA Decay

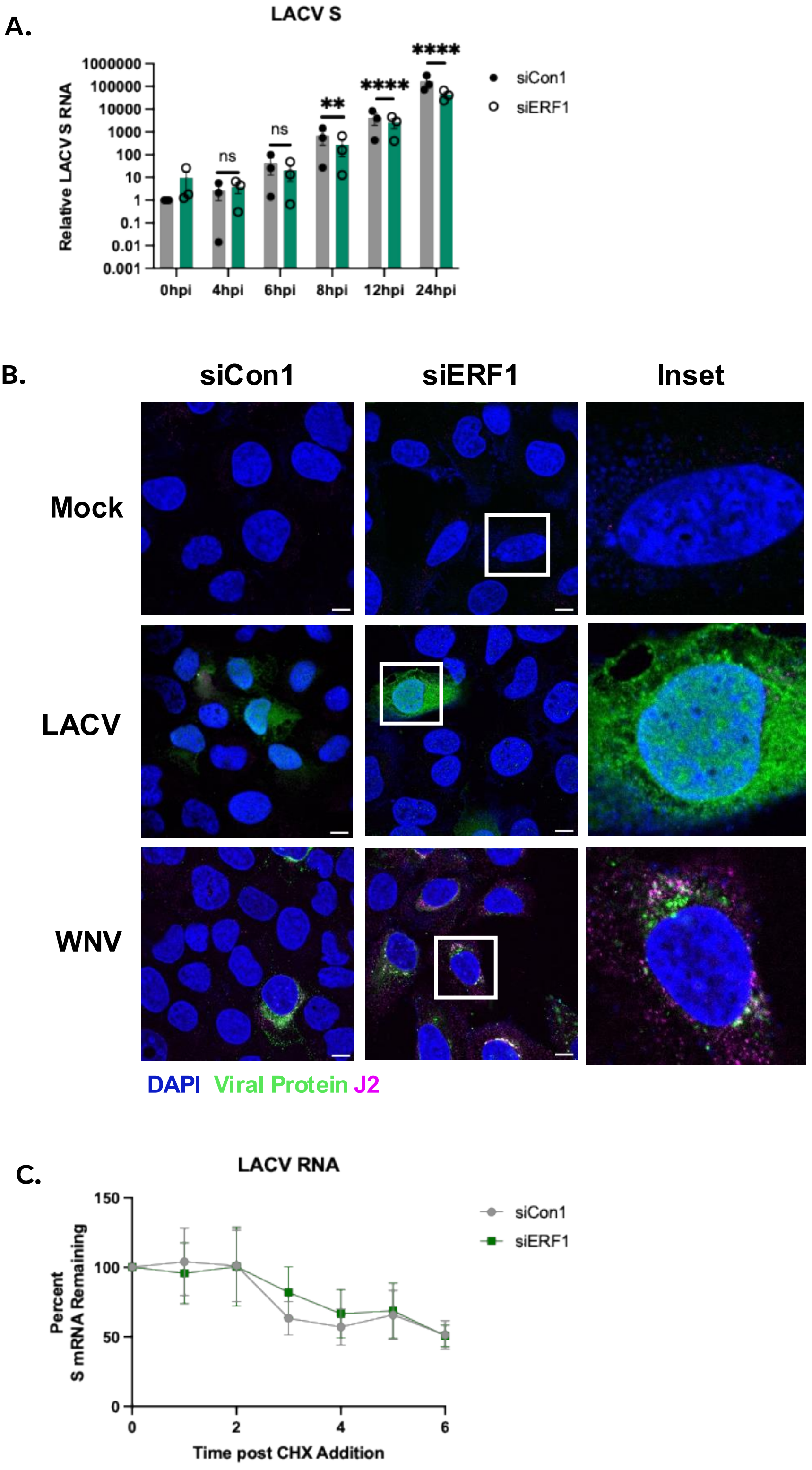

Supplementary Figure 3: NMD Does Not Impact LACV Infection

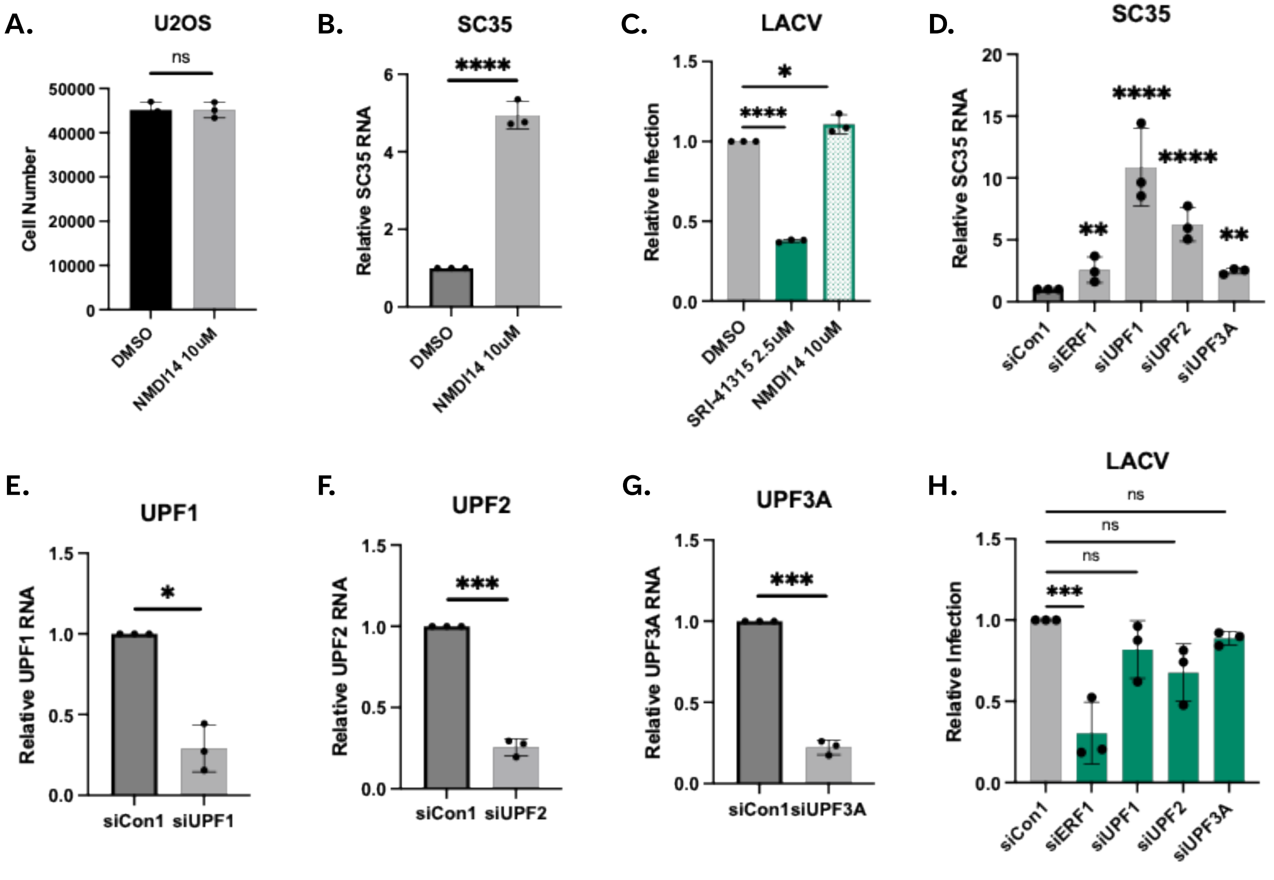

Supplementary Figure 4: eRF1 is Required for LACV ΔNSs Infection

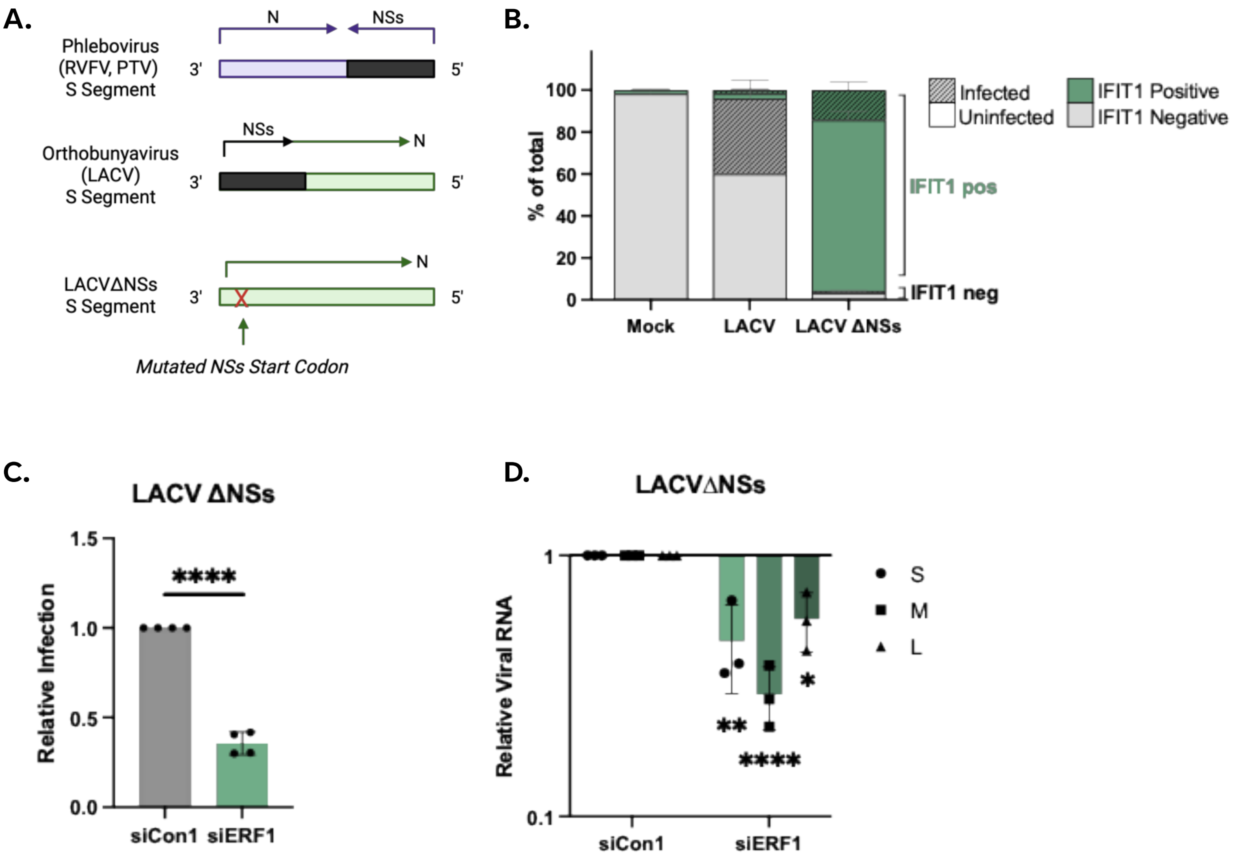

Supplementary Figure 5: NMD Does not Impact JCV'61 Infection

A.

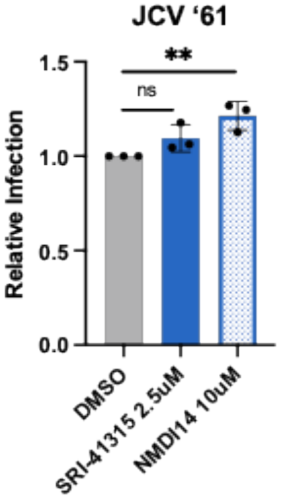

B.

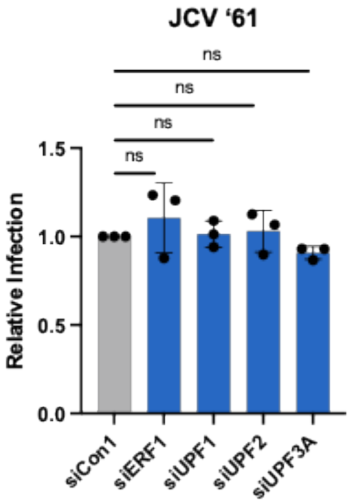

Supplementary Figure 6: NSm Alignment for 16 CSG Viruses

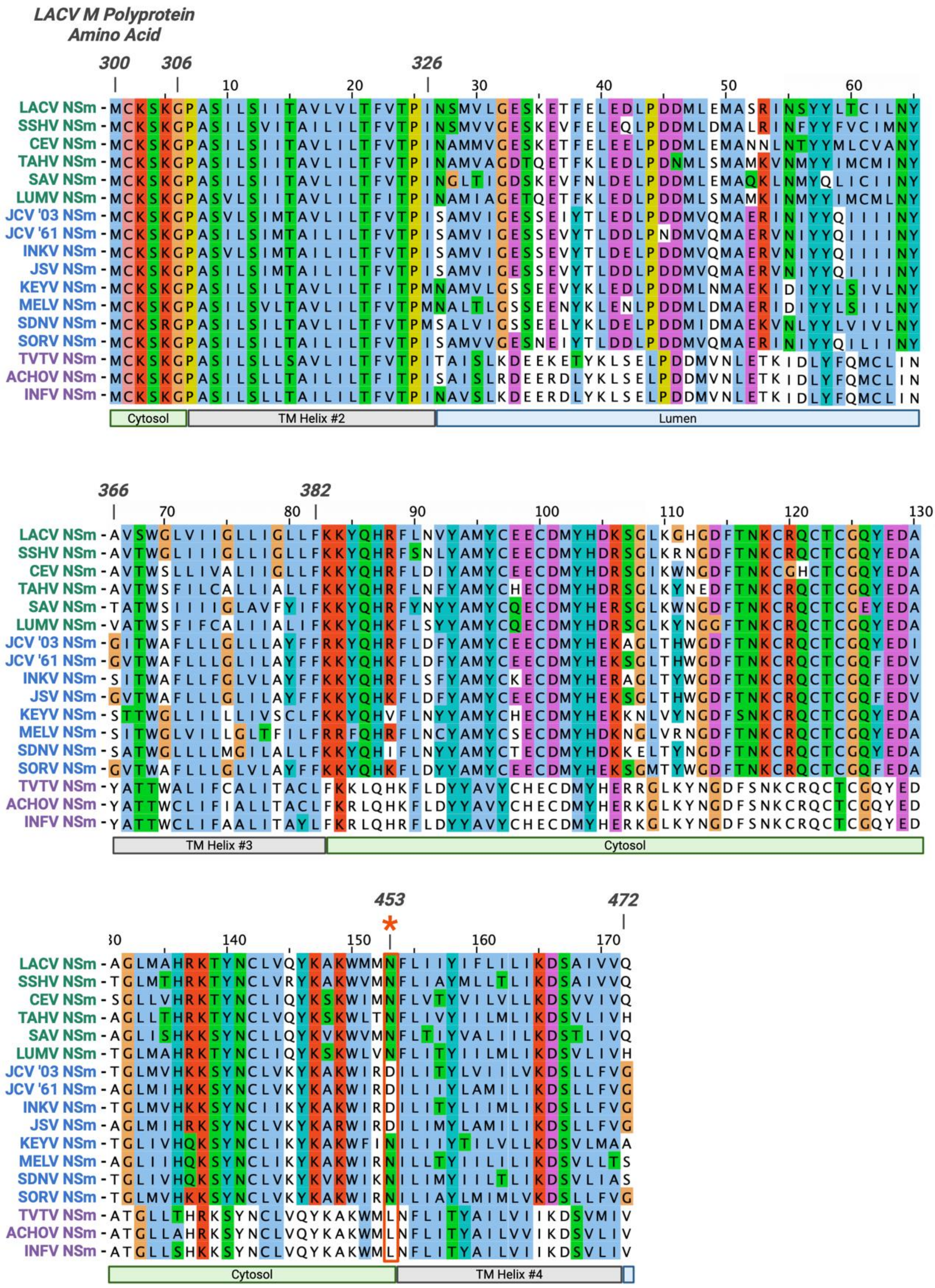

Supplementary Figure 7: LACV NSm AlphaFold Prediction

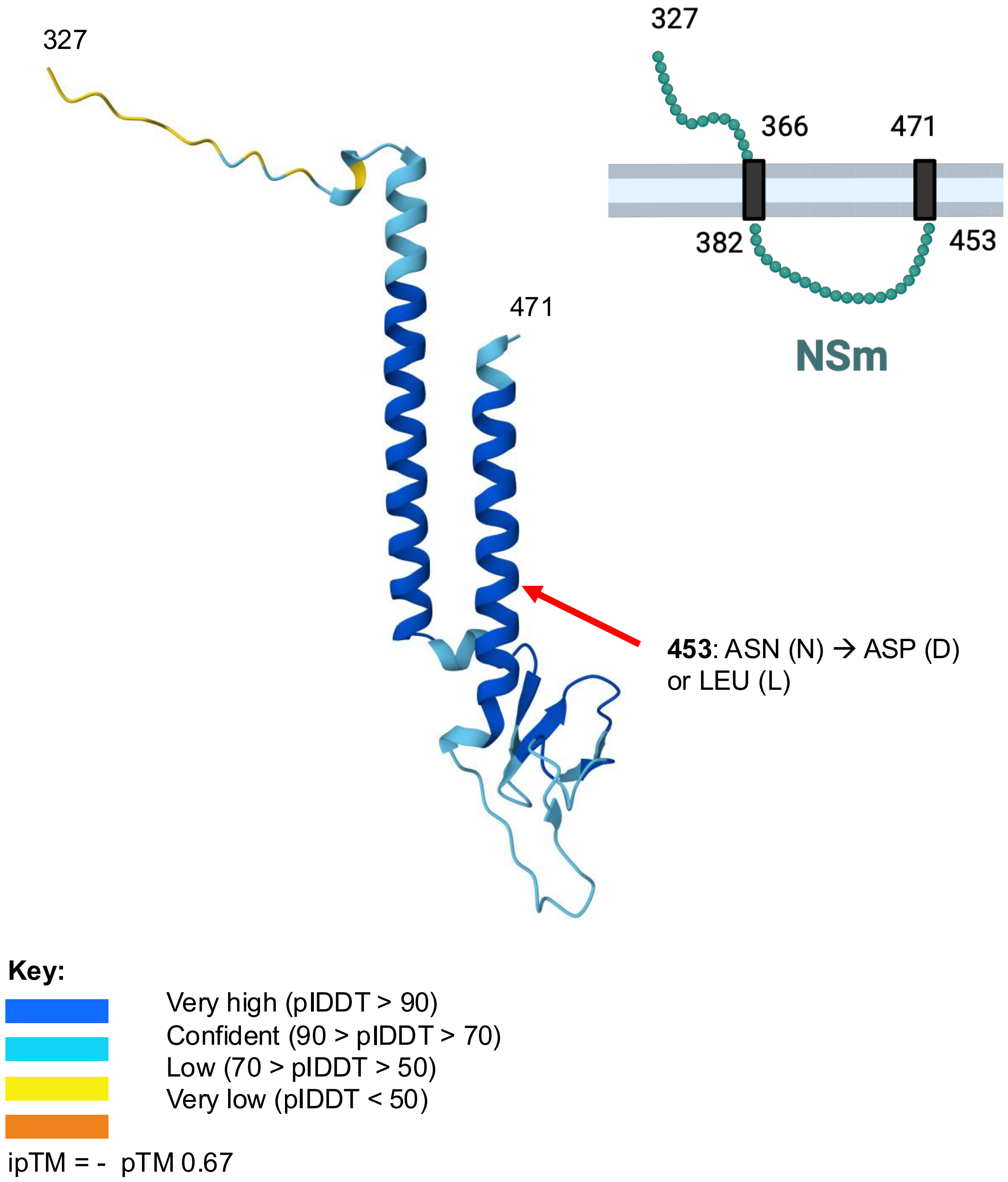

Supplementary Figure 8: Phylogenetic Trees

A. Bunyavirales Class – L Protein

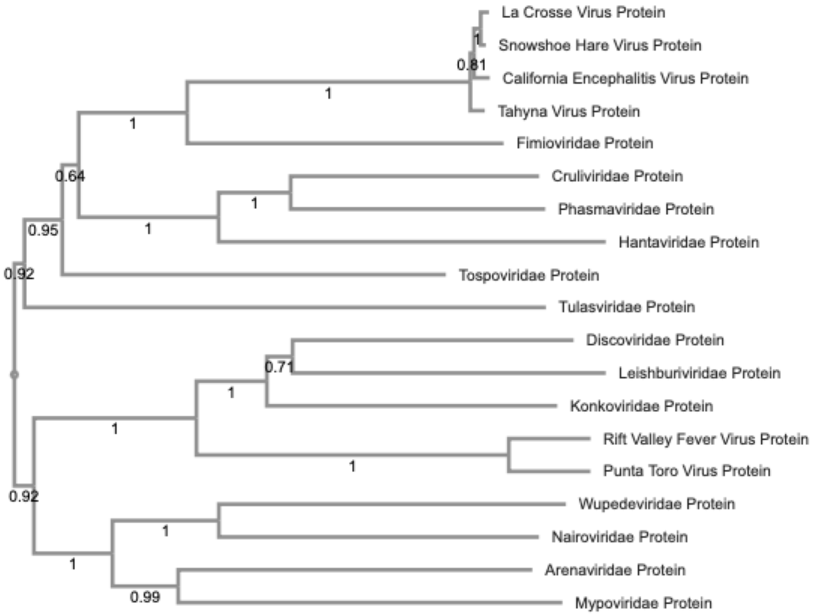

B. CSG Viruses – M Protein

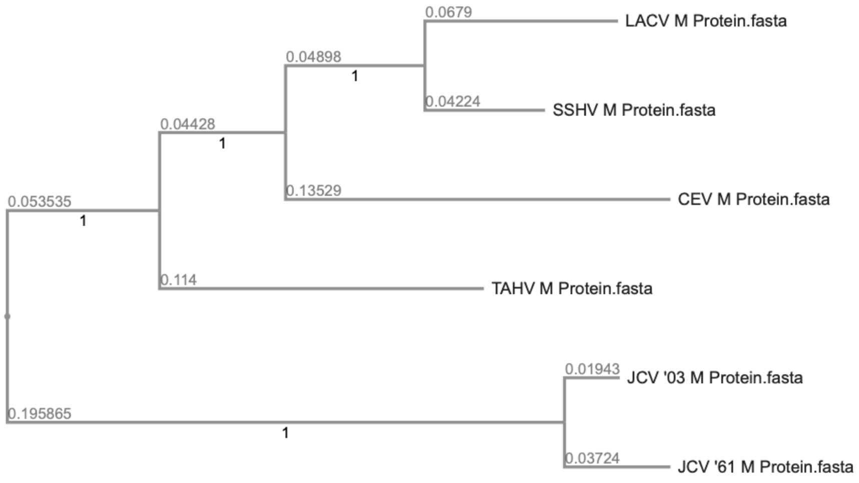

Supplementary Figure Legends

Supplementary Figure 1: Cell Viability and Knockdown Validation

(A) U2OS cells transfected with siCon1 or siDeath. After 72 hours, cell number was measured by automated microscopy. Mean ±SD shown for n=4. **(B)** Cell number results of the screen plotted as percent of control. SMU1 (blue) and EIF4A3 (orange) displayed greater than 50% cytotoxicity and were not selected for further study. **(C, D)** U2OS cells were transfected with siCon1 or siERF1 for 72 hours **(C)** eRF1 RNA were measured by RT-qPCR with GAPDH used as a control. Mean ±SD shown for n=3. **(D)** eRF1 protein levels were measured by immunoblot with tubulin as a control. A representative image is shown, n=2. **(E)** U2OS cells transfected with the indicated siRNAs for 72 hours. Cell number was measured by automated microscopy. Mean ±SD shown for n=3. **(F, G)** U2OS cells were mock infected or infected with LACV (MOI 0.1) for 24 hours. **(F)** eRF1 RNA levels were assessed by RT-qPCR with GAPDH used as a control. Mean ±SD shown for n=3. **(G)** eRF1 protein levels were assessed by immunoblot. A representative blot is shown for n=2. **(H, I)** U2OS cells transfected with the indicated siRNAs for 72 hours. **(H)** eRF3a **(I)** eRF3b RNA was measured by RT-qPCR with GAPDH used as a control. Mean ±SD shown for n=3. **(J)** U2OS cells were treated with DMSO or SRI-41315 at the indicated concentrations for 20 hours. Cell number was assessed by automated microscopy. Mean ±SD shown for n=3. **(K)** HFF cells were treated with DMSO or SRI-41315 3uM for 20 hours. Cell number was assessed by automated microscopy. Mean ±SD shown for n=3. Dots represent individual experiments. Statistical

analyses were performed using one-way ANOVA with Dunnett's multiple comparison test (G, H, I, J) and Student's (unpaired, two-tailed) t-test (A, C, E, K). *p<0.05, **p<0.01, ***p<0.001, ****p<0.0001, ns=not significant.

Supplementary Figure 2: eRF1 Depletion Does Not Induce LACV RNA Decay

(A) U2OS cells were transfect with siCon1 or siERF1. 72 hours later, infected with LACV (MOI 0.1) at 4ºC for 1 hour, before washing and replacing with media. Samples collected at the indicated time points post infection. LACV S RNA was measured by RT-qPCR with GAPDH as a control. Mean ±SEM shown for n=3. Y axis on a log 10 scale. **(B)** U2OS cells were transfected with siCon1 or siERF1. After 72 hours, cells were mock infected or infected with LACV (MOI 1) or WNV (MOI 5) for 18 hours and subject to confocal microscopy. With insets shown. Viral protein (green) and dsRNA (red) and nuclei (blue). Representative images shown for n=2; Magnification 63x; Scale bar is 10μm. **(C)** U2OS cells were transfect with siCon1 or siERF1. After 72 hours, cells were infected with LACV (MOI 0.1) for 18 hours, treated with 50ug/mL of cycloheximide (CHX). Samples were collected at the indicated time points. LACV S mRNA was measured by RT-qPCR with GAPDH as a control. Mean ±SD shown for n=3. Dots represent individual experiments. Statistical analyses were performed using one-way ANOVA with Dunnett's multiple comparison test (A) and a two-way ANOVA with Šídák's correction for multiple comparisons (C). **p<0.01, ****p<0.0001; ns=not significant.

Supplementary Figure 3: NMD Does Not Impact LACV Infection

(A, B) U2OS cells were treated with DMSO or NMDI14 10uM for 20 hours. (A) Cell number was measured by automated microscopy. Mean ±SD shown for n=3. (B) SC35 RNA was measured by RT-qPCR with GAPDH as a control. Mean ±SD shown for n=3. (C) U2OS cells were treated with DMSO, SRI-41315 at 2.5uM, or NMDI14 at 10uM for 20 hours before infecting with LACV (MOI 1) for 24 hours. Relative infection was measured by automated microscopy. Mean ±SD shown for n=3. (D, E, F, G) U2OS cells were transfected with the indicates siRNAs for 72 hours (D) SC35 RNA (E) UPF1 RNA (F) UPF2 RNA (G) UPF3A RNA was measured by RT-qPCR with GAPDH as a control. Mean ±SD shown for n=3. (H) U2OS cells were transfected with the indicated siRNAs for 72 hours before infecting with LACV (MOI 1) for 24 hours. Relative infection was measured by automated microscopy. Mean ±SD shown for n=3. Dots represent individual experiments. Statistical analyses were performed using Student's (unpaired, two-tailed) t-test (A, B, E, F, G) and one-way ANOVA with Dunnett's multiple comparison test (C, D, H). *p<0.05, **p<0.01, ***p<0.001, ****p<0.0001, ns=not significant.

Supplementary Figure 4: eRF1 is Required for LACV ΔNSs Infection.

(A) Schematic of the S segment coding strategy for *Orthobunyavirus* genus in green, the *Phlebovirus* genus in purple, and the LACV ΔNSs strain. **(B)** U2OS cells were infected with LACV (MOI 0.1) or LACV ΔNSs (MOI 3.33) for 24 hours. Infection and IFIT1 were analyzed by automated microscopy. Mean ±SD shown for n=3. **(C, D)** U2OS

cells were transfected with siCon1 or siERF1. 72 hours after transfection infected with LACV ∆NSs (MOI 3.33) for 24 hours. **(C)** Relative infection was measured by automated microscopy. Mean ±SD shown for n=3. **(D)** LACV S, M, and L RNA was measured by RT-qPCR with GAPDH as a control. Mean ±SD shown for n=3. Dots represent individual experiments. Statistical analyses were performed using one-way Student's (unpaired, two-tailed) t-test (C), and two-way ANOVA with Šídák's correction for multiple comparisons (D). *p<0.05, **p<0.01, ****p<0.0001.

Supplementary Figure 5: NMD Does Not Impact JCV'61 Infection

(A) U2OS cells were treated with DMSO, SRI-41315 at 2.5uM, or NMDI14 at 10uM for 20 hours before infecting with JCV (MOI 1.8) for 24 hours. Relative infection was measured by automated microscopy. Mean ±SD shown for n=3. **(B)** U2OS cells were transfected with the indicated siRNAs for 72 hours before infecting with JCV'61 (MOI 1.8) for 24 hours. Relative infection was measured by automated microscopy. Mean ±SD shown for n=3. Dots represent individual experiments. Statistical analyses were performed using one-way ANOVA with Dunnett's multiple comparison test (A, B). *p<0.05, **p<0.01, ***p<0.001, ****p<0.0001, ns=not significant.

Supplementary Figure 6: NSm Alignment for 16 CSG Viruses

NSm alignments with Clustal color scheme. The grey text and numbers indicate the residues from LACV corresponding to the 1441 amino acid from the LACV M

polyprotein (i.e. 300-472). CEV complex viruses, green; MELV complex viruses, blue; TVTV complex viruses, purple. The notable amino acid difference is noted in red with an asterisk. The predicted topology of NSm: transmembrane helixes in grey, cytosolic facing in green, lumen facing in blue.

Supplementary Figure 7: LACV NSm AlphaFold Prediction

To visualize the cytoplasmic portion of NSm, as predicted by DeepTMHMM (Figure 5D), amino acids 371 to 471 from LACV M polyprotein analyzed by Alphafold. As expected, the TMs form helices. The notable amino acid from Figure S5 and changes are noted with a red arrow. The colors denoted in the key are the level of confidence output by the software.

Supplementary Figure 8: Phylogenetic Trees

(A) *Bunyaviricites* phylogenetic tree based on large (L) protein amino acid sequences of the 15 families. The phylogenetic tree was constructed using L segment amino acid sequences from Genbank and assembled using BV.BRC.org Protein Tree builder. Tree segments are to scale. The numbers indicate the confidence at each node. **(B)** Phylogenetic tree of LACV, SSHV, CEV, TAHV, and JCV' 03 and JCV '61. Assembled based on the M segment amino acid sequence using software provided by BV-brc.org. Tree branches to scale. The numbers indicate the confidence at each node.

Supplementary Table 1: siRNA Screen Data

Supplementary Table 2: TCID50 Titers for all Recombinant Viruses

TCID50 in U2OS cells. Viruses in **bold** are sensitive to eRF1 depletion.

Supplementary Table 3: DeepTMHMM Predictions

Protein topology predictions for LACV, JCV ’61, and the r/LACV-J-NSm viruses. Accession numbers are noted for LACV and JCV ’61, as well number of transmembrane domains and amino acid length of the polyprotein are noted for each virus.

Supplementary Table 4: Stop Codon Analysis

Analysis of the termination region for the S, M, and L segment for LACV, SSHV, CEV, TAHV, JCV ’03, and JCV ’61.

Supplementary Table 5: Primers

Supplementary Table 6: siRNA Sequences

Supplementary Table 7: Viral Species and Accession Numbers

Viral Species and GenBank accession numbers. Viruses in Bold are used in this study.

Table S1: siRNA Screen Data

Gene Symbol	POC_%Positive_ Ambion	POC_%Positive_ Dharmacon	POC_TotalCells_ Ambion	POC_TotalCells_ Dharmacon
DDX1	179.1	119.7	112.0	107.1
DDX10	298.6	88.6	100.2	95.5
DDX23	68.0	75.0	52.5	87.5
DDX41	38.8	72.2	92.4	102.7
DDX51	126.8	107.7	118.4	104.2
DDX60	151.3	106.4	133.5	110.6
GINS2	82.0	92.9	110.5	111.6
SMU1	2.6	16.8	26.8	37.7
ZNFX1	74.3	100.3	117.6	102.9
EIF4A1	88.2	152.6	71.7	74.3
DDX11	63.4	163.2	136.9	108.7
DDX24	171.9	97.1	40.0	69.0
DDX42	75.7	106.2	94.0	96.0
DDX52	83.3	52.1	105.8	102.4
DDX60L	114.1	123.4	118.4	114.3
GNB2L1	0.1		83.4	
STAT1	55.5	90.3	117.7	108.7
TMPRSS2	98.8	112.0	117.6	107.1
EIF4A2	170.7	109.3	86.1	112.2
DDX17	102.7	84.9	114.4	103.6
DDX25	34.9	103.9	126.4	109.9
DDX43	56.3	130.2	95.1	108.1
DDX53	139.4	90.8	115.3	105.3
AHR	132.9	86.8	115.8	111.6
GNB4	81.1	125.7	127.6	107.3
TNFSF10	76.1	79.8	101.8	107.9
FRAP1	99.3		102.6	
DDX3X	116.2	147.7	77.3	91.2
DDX18	94.0	140.5	73.7	89.2
DDX27	240.7	122.6	91.6	100.8
DDX46	63.2	68.0	88.1	99.9
DDX54	159.7	153.2	87.1	90.4
BAZ1A	138.8	119.8	118.5	111.2
HSPE1	83.3	74.4	85.2	99.7
TRAFD1	113.2	103.1	100.0	106.8
RICTOR	88.0	97.6	108.9	106.0
DDX3Y	70.3	105.9	84.8	111.2
DDX19A	56.2	102.3	126.2	109.3
DDX28	55.6	89.7	118.1	107.2
DDX47	108.7	108.2	104.0	96.0
DDX55	108.3	62.5	120.6	111.3
CDC123	59.5	97.4	128.3	110.8
IKZF3	121.8	79.4	135.4	110.8
TRIM22	69.7	105.8	97.0	110.4
RPTOR	138.7	99.6	108.7	110.3

DDX4	199.6	94.9	81.6	109.8
DDX19B	28.9	85.3	90.3	83.5
DDX31	84.1	99.9	122.7	106.8
EIF4A3	11.8	13.7	9.7	19.4
DDX56	186.8	148.3	53.5	82.2
DBR1	142.6	78.3	94.8	98.2
LSM2	36.5	32.0	75.8	75.2
TTLL7	75.6	106.6	109.9	111.2
CSNK2A1	97.7	122.1	117.3	113.4
DDX5	64.9	159.8	97.8	95.2
DDX20	167.7	108.9	104.1	109.7
DDX39A	149.0	97.7	118.9	108.7
DDX49	102.9	100.5	104.7	94.4
DDX58	83.2	83.0	130.2	111.9
EIF2S1	48.0	83.3	94.8	107.0
RSRC2	51.4	60.2	107.1	111.6
TXNL4A	76.4	83.0	95.9	96.0
CSNK2A2	75.1	129.5	118.5	108.5
DDX6	254.0	199.6	104.8	88.9
DDX21	83.9	143.4	61.9	81.3
DDX39B	84.9	86.2	93.8	85.8
DDX50	105.0	79.5	110.0	83.7
DDX59	134.9	163.7	115.1	107.1
ETF1	13.6	16.8	68.4	66.9
SIK3	61.4	54.8	124.5	105.4
WARS	111.8	153.6	86.4	101.9
CSNK2B	116.7	103.8	119.5	117.0
RACK1		4.8		84.0
MTOR		85.2		107.2

Table S2: TCID50 Titers for All Recombinant Viruses

**Virus**	**TCID50 Titer in U2OS Cells**
**LACV**	1.26E+06
JCV '03	3.65E+06
JCV '61	5.62E+05
**r/LACV**	4.22E+05
r/JCV'03	1.47E+06
**r/JLL**	1.00E+06
**r/JLJ**	1.00E+07
r/JJL	7.50E+05
**r/LLJ**	1.00E+04
r/LACV-J-NSm	3.16E+05

TCID50 in U2OS cells. Viruses in bold are sensitive to eRF1 depletion.

Table S3: DeepTMHMM Predictions

LACV M Polyprotein: YP_010839410.1, # of amino acids: 1441**,** # of TM Domains: 5

JCV '61 M Polyprotein: YP_009666885.1, # of amino acids: 1444**,** # of TM Domains: 5

LACV M Polyprotein with J'61 172 NSm: # of amino acids: 1441**,** # of TM Domains: 5

**Feature**	**Amino Acid: Start-End**
Signal Peptide	1-12
Outside	13-200
TM helix	201-221
Inside	222-305
TM helix	306-326
Outside	327-365
TM helix	366-382
Inside	383-452
TM helix	453-471
Outside	472-1395
TM helix	1396-1416
Inside	1417-1441
**Feature**	**Amino Acid: Start-End**
Signal Peptide	1-15
Outside	16-202
TM helix	203-223
Inside	224-307
TM helix	308-328
Outside	329-361
TM helix	362-384
Inside	385-454
TM helix	455-475
Outside	476-1398
TM helix	1399-1419
Inside	1420-1444
**Feature**	**Amino Acid: Start-End**
Signal Peptide	1-12
Outside	13-200
TM helix	201-221
Inside	222-305
TM helix	306-326
Outside	327-361
TM helix	362-382
Inside	383-453
TM helix	454-474
Outside	475-1395
TM helix	1396-1416
Inside	1417-1441

##gff-version 3

Table S4: Stop Codon Analysis

Virus	Segment/ORF	- 10	Stop Codon	+ 10
LACV	NSs	U CUG GGU AUU	UAG	CCA GAU GGG U
1960	N	C AGG CUU CCA	UAA	AUA UGG CAU G
	M	G AAA AUU AGA	UAG	GGG AUC UAU G
	L	A GAG UUU GAU	UAG	UAG UUA UGA G
SSHV	NSs	U CAG GAU AUU	UAG	CCA GAU GGG U
Montana 1959	N	C AGG CUC CCA	UAA	GUA AGG AGG U
	M	G AAG AUU AGG	UAA	GAG GGU GCA G
	L	A GAA UUU GAU	UAG	UUG AUC AGA A
CEV	NSs	A GAU GGG UCC	UAG	AGC AAU ACA A
BFS-283	N	A AAG CUG CCA	UAA	GCU CUA GCA G
	M	G AAA AUA AGA	UAA	UAA GCA AAA G
	L	U GAU UUU GAG	UAA	GUA UUU CAU U
TAHV	NSs	A GAU GGG UGC	UAG	AGC AGU UCA A
Czechoslovakia '92	N	C AGA CUC CCA	UAA	AUG GGA UUA A
	M	G AAA AUU AGG	UAA	GGA GCA UAG C
	L	A GAA UUC GAU	UAA	ACA AUA GAG C
JCV '03	NSs	U CCG GAU AUC	UAG	CCA GAU GGG U
2003 Isolate	N	A AAA CUG CCU	UGA	ACA AAU GCA A
	M	G AAA AUA AGG	UAA	UCC AGA UAA G
	L	AGA UUU UAG U	UAG	AUA AAG UGA G
JCV '61	NSs	U CAG GAU AUC	UAG	CCA GAU GGG U
1961 Isolate	N	A AAG UUG CCA	UAA	ACA AAA AUA G
	M	G AAG AUA AGA	UGA	UUC ACA UCA G
	L	A GAU UUC AGU	UAA	GCA GGG UGA G

Analysis of the termination region for the S, M, and L segment for LACV, SSHV, CEV, TAHV, JCV ’03, and JCV ’61.

Table S5: Primers

Target	Forward	Reverse
GAPDH	ACCAAATCCGTTGACTCCGACCTT	TGCACAGTCAGCCGCATCTTCTTT
ETF1 (eRF1)	ACTGGGCCCTTAGGGAGG	TGCCACTCGTGAAATCTGGT
GSPT1 (eRF3a)	GAGGAGGAAGAGGAAATCCC	TCCTTTTGTCAACCATTCCA
GSPT2 (eRF3b)	CACTGCCACACAGCAAGTTC	TTGACGTTGAGCTTACGGCT
UPF1	TGAAGGAGTCCCAGACTCAA	ACTAAATCCTCATTACCAGAGTCAG
UPF2	TCACCAAAAATGACACACTGC	AGTACACTGGTCCTCAGGTG
UPF3A	GACTGGCAAACAAGGACCG	ACTCACTCTGCCTCTTCCCT
SC35	CGGTGTCCTCTTAAGAAAATGATGTA	CTGCTACACAACTGCGCCTTTT
LACV S	GCTGAGTCTAATGGTGTAGGATG	TGGTCAGCGGGTAGAATTTG
LACV M	CCTGCCTAGAGACTGAGAGTAT	GAGTTGCAATGTTGGTGTAAGG
LACV L	GCAAAGCAAAGGGCCTAAAG	GTGGGTAGAGGATAGGGTCTAA
SSHV S	ACCCCATTGCCGAGTCAAAT	AATTCCAATGGTCAGCGGGT
CEV S	CCACCGCATCTCAGGATACC	TGACTCCGCAATTGGGTTGA
TAHV S	CAGGTGGAGGTCGTCAATAAT	AGCACCCATCTAGCCAAATAC
RVFV S	CAAGCAGTGGACCGCAATGAGA	GGGCTTGTTGCCACGAGTTAGA
PTV S	CCAGCAGTTATCCTAGGGCG	ATTGGTCGCTCAAAGCTGGA
JCV S	AGCAAAAGCCAAAGCTGCTC	CCTGAAAGCCGATGGATGGT
LACV / JCV r Viruses	TGGATGGTAAGATCGTTGTTACCAA	TTTGGAGAGTGGCAGGTGGAG
**LACV/JCV ’61 NSm Recombinant Virus – Cloning Primers**		
LACVpM_overhang	CGTTATTATTCGTTGGA**GCTGCTGGAACTGACTTCACCACC**	CCTTTTGATTTGCACATAAC**TCTGGCAGCTCTTAGGC**
J61_NSm_insert	**CTAAGAGCTGCCAGA**GTTATGTGCAAATCAAAAGGACCAG	**GTCAGTTCCAGCAGC**TCCAACGAATAATAACGAATCTTTG

Bolded sequence corresponds to backbone. Underlined sequence corresponds to JCV/61 insert

Table S6: siRNA Sequences

Oligo Name	Sequence (5' - 3')	Overhang	Sequence (5' - 3')	Overhang
siETF1_A	GGAUAUAAUGAGAUAUGUU	(dT)(dT)	AACAUAUCUCAUUAUAUCC	(dA)(dG)
siETF1_B	GUGGAACAAUUGUAACAGA	(dT)(dT)	UCUGUUACAAUUGUUCCAC	(dA)(dG)
siGSPT1_A	GAUUACCGUUUAUUCCAUA	(dT)(dT)	UAUGGAAUAAACGGUAAUC	(dC)(dA)
siGSPT1_B	GAACUUCAAUAGAUCAGUU	(dT)(dT)	AACUGAUCUAUUGAAGUUC	(dG)(dG)
siGSPT2_A	GCCGUUACCAUGGAACUUU	(dT)(dT)	AAAGUUCCAUGGUAACGGC	(dT)(dG)
siGSPT2_B	CAGAACCUGUUGUAGAAAA	(dT)(dT)	UUUUCUACAACAGGUUCUG	(dA)(dA)
siUPF1_A	CAGCGGAUCGUGUGAAGAA	(dT)(dT)	UUCUUCACACGAUCCGCUG	(dC)(dA)
siUPF1_B	CAACGGACGUGGAAAUACU	(dT)(dT)	AGUAUUUCCACGUCCGUUG	(dC)(dA)
siUPF2_A	GCAACGAAGUGGUGAAUCU	(dT)(dT)	AGAUUCACCACUUCGUUGC	(dT)(dG)
siUPF2_B	GCUCGGAAUUUUUAUGAGA	(dT)(dT)	UCUCAUAAAAAUUCCGAGC	(dA)(dT)
siUPF3A_A	GCAGCUGUAUGAUCCAGGA	(dT)(dT)	UCCUGGAUCAUACAGCUGC	(dA)(dA)
siUPF3A_B	CCACACCUCUUUUGGAAUA	(dT)(dT)	UAUUCCAAAAGAGGUGUGG	(dT)(dT)

Table S7: Viral Species and Accession Numbers

Virus Family	Viral Species	Genebank Accession Number – L Segment and L Protein	Genebank Accession Number – M Segment and M Protein	Genebank Accession Number – S Segment
Cruliviridae	Chinese mitten crab virus 1	NC_078281.1, YP_010839952.1		
Tospoviridae	Melon severe mosaic tospovirus	NC_033834.1, YP_009346017.1		
**Peribunyaviridae**	**La Crosse Virus 1960**	NC_077810.1, YP_010839411.1	NC_077809.1, YP_010839410.1	NC_077808.1
**Peribunyaviridae**	**Snowshoe Hare Virus Montana 1959**	NC_055196.1, YP_010085080.1	NC_055197.1, YP_010085081.1	NC_055198.1
**Peribunyaviridae**	**California Encephalitis Virus BFS-283**	NC_055119.1, YP_010084300.1	NC_055118.1, YP_010084299.1	NC_055117.1
**Peribunyaviridae**	**Tahyna Virus Czechoslovakia '92**	HM036210.1, ADQ08670.1	HM036209.1, ADQ08658.1	HM036208.1
Fimioviridae	Common oak ringspot-associated virus	NC_078852.1, YP_010840591.1		
Hantaviridae	Thottapalayam Virus	NC_010707.1, YP_001911124.1		
Phasmaviridae	Wuhan Mosquito Virus 1	NC_031307.1, YP_009305130.1		
Tulasviridae	Tulasnella bunyavirales-like virus 1	NC_076861.1, YP_010800386.1		
Konkoviridae	Tulip Streak Virus	LC571987.2, BCK51609.1		
Leishburiviridae	Leptomonas moramango Virus	NC_055204.1, YP_010085090.1		
**Phenuiviridae**	**Rift Valley Fever Virus ZH-548**	NC_014397.1, YP_003848704.1		
**Phenuiviridae**	**Punta Toro Virus Balliet**	KR912212.1, ALL45372.1		
Discoviridae	Rice dwarf-associated bunya-like virus	ON366507.1, UTJ93941.1		
Arenaviridae	Mopeia virus AN20410	NC_006574.1, YP_170708.1		
Mypoviridae	Hubei myriapoda virus 5	NC_033761.1, YP_009345128.1		
Wupedeviridae	Wuhan Millipede Virus 2	NC_043500.1, YP_009666319.1		
Nairoviridae	Crimean-Congo hemorrhagic fever virus	NC_005301.3, YBU56725.1		
**Peribunyaviridae**	**Jamestown Canyon Virus 2003 Strain**	HM007355.1, YBB96240.1	HM007354.1, YBB96208.1	HM007353.1
**Peribunyaviridae**	**Jamestown Canyon Virus 1961 Strain**	NC_043559.1, YP_009666884.1	NC_043560.1, YP_009666885.1	NC_043558.1
Peribunyaviridae	San Angelo Virus		NC_043638.1	
Peribunyaviridae	Lumbo Virus		NC_043630.1	
Peribunyaviridae	Inkoo Virus		KT288270.1	
Peribunyaviridae	Jerry Slough Virus		KX817319.1	

Peribunyaviridae	Keystone Virus		NC_043627.1	
Peribunyaviridae	Melao Virus		NC_043633.1	
Peribunyaviridae	Serra do Navio Virus		NC_043639.1	
Peribunyaviridae	South River Virus		KX817337.1	
Peribunyaviridae	Trivittatus Virus		NC_055193.1	
Peribunyaviridae	Achiote Virus		KY555809.1	
Peribunyaviridae	Infirmatus Virus		NC_077936.1	
